# RNA Splicing Aberrations in Hereditary Cancer: Insights from Turkish Patients

**DOI:** 10.3390/cimb46110790

**Published:** 2024-11-20

**Authors:** Seda Kilic, Ozge Sukruoglu Erdogan, Seref Bugra Tuncer, Betul Celik Demirbas, Zubeyde Yalniz Kayim, Hulya Yazici

**Affiliations:** 1 Cancer Genetics Department, Division of Basic Oncology, Institute of Oncology, Istanbul University, 34093 Istanbul, Türkiye; ozge.erdogan@istanbul.edu.tr (O.S.E.); seref.tuncer@istanbul.edu.tr (S.B.T.); betul.celik@istanbul.edu.tr (B.C.D.); zubeyde.yalniz@istanbul.edu.tr (Z.Y.K.); hulyayazici67@gmail.com (H.Y.); 2 Medical Biology and Genetics Department, Medical Faculty, Istanbul Health and Technology University, 34275 Istanbul, Türkiye

**Keywords:** mRNA, splicing error, variant of clinical relevance, functional, cancer, Turkish

## Abstract

The process of RNA splicing is fundamental in contributing to proteomic diversity and regulating gene expression. Dysregulation of splicing is associated with various human disorders, including cancer. Through functional studies, this study sought to examine the potential impact of seven variants within six inherited cancer-related genes on RNA splicing patterns in Turkish cancer patients. Upon detecting variants using Next-Generation Sequencing (NGS), we used Reverse Transcriptase Polymerase Chain Reaction (RT-PCR) and Sanger sequencing to elucidate the effects of these variants on splicing. Three of the seven variants demonstrated no discernible effect on RNA, while four exhibited pathogenic characteristics. Specifically, the variants *APC* c.532-1G>A rs1554072547, *BRCA1*c.4358-3A>G rs1567779966, *BRCA2*c.7436-1G>C rs81002830 and *MSH3*c.1897-1G>A rs1744149615 were identified as pathogenic, while the variants *BLM*c.4076+4T>G rs183176301, *RB1*c.2489+2T>C rs1555294636 and *RB1*c.1050-2A>G rs? were found to be benign from a splicing perspective. These findings highlight the importance of verifying the precise consequences of splice-site variants through experimental analysis, given their potential implications for genetic disorders and cancer predisposition. This research contributes to the understanding of splice-site variants in inherited cancer predisposition, particularly among Turkish cancer patients. It emphasizes the necessity for further exploration into the mechanisms and functional consequences of alternative splicing for potential therapeutic interventions in cancer.

## 1. Introduction

The human genome is a complex entity consisting of protein-coding and non-coding sequences, representing only a small fraction of the DNA [1]. Recent studies estimate the presence of approximately 20,000 genes in the human genome, a relatively low number compared to the diverse range of proteins observed in humans [2]. The discrepancy highlights the existence of intricate regulatory pathways, including post-translational modifications and alternative splicing, which contribute significantly to protein diversity [3].

Alternative splicing, a well-established process, allows for the generation of multiple transcripts from a single mRNA precursor, thus playing a crucial role in expanding proteomic diversity [4]. Nearly 94% of human transcripts undergo alternative splicing, underscoring its prevalence and essential role in gene expression control [5]. RNA-binding proteins regulate alternative splicing by interacting with cis-regulatory elements near splice sites, thereby modulating splice-site usage [6]. Alternative splicing is vital for various biological processes, such as spermatogenesis, and has been implicated in conditions like male infertility [7]. Moreover, it is closely associated with the progression of numerous human diseases, including cancer, with disruptions in splicing often contributing to disease development [8]. Over 50% of genetic disorders are associated with abnormalities in the splicing process.

Understanding the impact of DNA variants on splicing mechanisms is crucial, as it provides valuable insights into identifying pathogenic variants and defining potential therapeutic targets. This study aims to investigate the effects of variants detected in NGS analyses of cancer patients who applied to our clinic, focusing on variants predicted to be pathogenic by in silico algorithms. Additionally, we explore how alterations in pre-mRNA splicing, particularly at exon/intron junctions, may disrupt gene function and affect protein structure and function. This study also aims to evaluate variants of unknown clinical significance using samples from cancer patients undergoing genetic testing.

The growing evidence highlighting the critical role of alternative splicing in cellular processes and disease onset emphasizes the importance of this study. By elucidating the effects of splicing alterations on gene function and protein structure, this study seeks to deepen our understanding of the impact of alternative splicing in cancer patients, potentially paving the way for targeted therapeutic interventions.

## 2. Materials and Methods

### 2.1. Patient Sample Collection and Processing

The inception of our study stemmed from genetic tests conducted on 1500 cancer patients visiting the Istanbul University Oncology Institute Cancer Genetics Department clinic between 2015 and 2019. This study adhered to the principles of the Declaration of Helsinki [9] and with ethical approval from the Istanbul University Faculty of Medicine Ethics Committee (2018/191). Subsequent to obtaining informed consent from the patients, peripheral blood samples were collected from the cancer patients. Exclusively samples from patients who had undergone genetic testing at our clinic were utilized in this study. Upon their initial visit for genetic testing, their family histories were recorded, and pedigrees were constructed.

### 2.2. DNA and RNA Isolation

DNA and RNA were isolated from peripheral blood samples using the AllPrep DNA/RNA Mini Kit (QIAGEN) in accordance with the provided instructions. The isolated DNA and RNA were visualized by %1 agarose gel electrophoresis and quantified using NanoDrop 2000c Spectrophotometer (Thermo Fisher Scientific, Waltham, MA, USA). RNA samples were stored in nitrogen tanks after isolation process until cDNA synthesis.

### 2.3. cDNA Preparation

The cDNA synthesis was carried out using the PrimeScript 1st Strand cDNA Synthesis Kit (Takara, Shiga, Japan), and the synthesized cDNA was employed for RT-PCR analysis.

### 2.4. RT-PCR Reaction and Sequencing

The primer sequences for the variants planned for investigation were designed using online programs Primer-BLAST [10] and Primer-Quest (https://eu.idtdna.com/pages/tools/primerquestst (accessed on 16 November 2024)). The sequences of the synthesized oligonucleotides can be found in Table 1.

### 2.5. PCR Product Purification and Sanger Sequencing

The DNA amplification process utilized the DreamTaq DNA Polymerase enzyme, and the resultant products underwent purification using the High Pure PCR Product Purification Kit from Sigma-Aldrich, Darmstadt, Germany. Subsequently, the Sanger sequencing reaction was executed by employing The GenomeLab Methods Development Kit (Beckman Coulter Inc., Brea, CA, USA), with the sequencing products then purified using a solution of glycogen, sodium EDTA, and sodium acetate in a ratio of 1:2:2, and 95% cold ethanol. Following this purification step, the Sanger sequencing reaction was performed using The GenomeLab Methods Development Kit (Beckman Coulter Inc., USA) and the GenomeLab GeXP Analysis System’s (Beckman Coulter Inc., USA) capillary electrophoresis device was employed to analyze and visualize the sequencing results.d. The acquired results were subjected to comparison with reference gene sequences (Gene Reference Numbers: *APC*: NM_000038.4, *BLM*: NM_000057, *BRCA1*: NM_007294.3, *BRCA2*: NM_000059.3, *MSH3*: NM_002439.4, *RB1*: NM_000321.2). The results were analyzed using the Genome Analyzer (GenomeLab GeXP Analysis System, Beckman Coulter Inc., USA) and the electropherogram image was reviewed by comparing it with the reference gene sequences on Alamut™ [11].

### 2.6. Splicing Analysis Using Bioinformatics Tools

In the process of conducting in silico analysis on variants identified as having potential splicing-related risks, a range of tools were used. Our study employed tools including SpliceSiteFinder-like [12], MaxEntScan [13], GeneSplicer [14], NNSPLICE [12], Human Splicing Finder [15], Rescue-ESE [16] and ESE-Finder [17] to predict alterations within splicing regions and to gauge the effects of variants within these regions. These in silico tools are fully integrated with Alamut^®^ Visual, and the assessments using these tools were conducted via Alamut^®^ Visual [11]. Additionally, insights derived from MutationTaster [18], SIFT [19], and g1000 [20] data were obtained through the utilization of Sophia DDM software (version 5.10, Sophia Genetics, Rolle, Switzerland) [21]. It is worth noting that databases such as HGMD [22], ClinVar [23], and LOVD [24] were utilized to trace previously reported publications and annotations associated with variants. A summary of the in silico tools and their respective predictions is provided in Table S1, which is included in the Supplementary Material due to the table size.

## 3. Results and Discussion

In our study, we evaluated the NGS panel results from 1500 patients who visited our clinic for genetic analysis. From these results, we focused on seven rare variants that were deemed potentially risky in terms of splicing, either due to their low frequency or their location within the gene, suggesting that they might coincide with splice donor or acceptor regions. The selected variants were as follows: *APC* c.532-1G>A rs1554072547, *BLM* c.4076+4T>G rs183176301, *BRCA1* c.4358-3A>G rs1567779966, *BRCA2* c.7436-1G>C rs886040939, *MSH3* c.1897-1G>A rs1744149615, and *RB1* c.2489+2T>C rs1555294636 and *RB1* c.1050-2A>G rs?. Initially, the variants were analyzed using in silico tools to predict their potential impact on splicing, followed by a comprehensive literature review to assess their reported pathogenicity. To experimentally evaluate the effect of these variants on mRNA and splicing, RNA was isolated from patients’ white blood cells and subsequently used to synthesize cDNA. RT-PCR was then performed using site-specific primer pairs, and the amplified products were sequenced via Sanger sequencing (the primer sequences used for RT-PCR and sequencing are listed in Table 1). The interpretation of the sequencing results provided insights into the possible consequences of these variants on mRNA splicing and transcript formation.

### 3.1. APC c.532-1G>A rs1554072547

**Figure 1:**
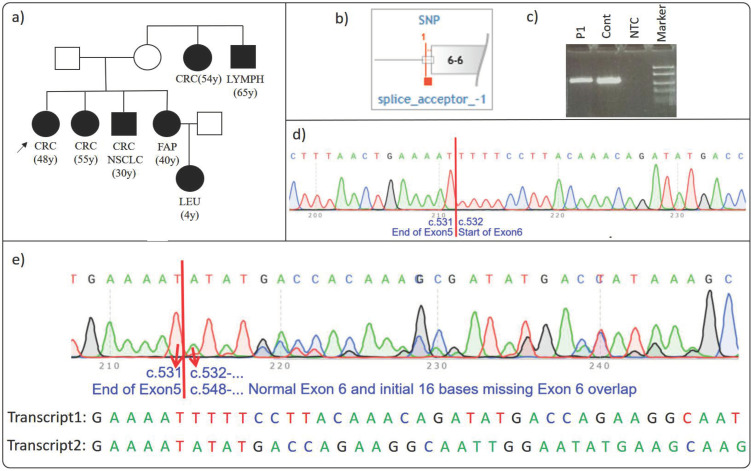
(**a**–**e**): Effect of *APC* c.532-1G>A on splicing. ((**a**) Proband’s pedigree. (**b**) Diagram showing the variant’s proximity to the relative exon and its location. (**c**) RT-PCR agarose gel electrophoresis: The relevant region was successfully amplified on proband’s cDNA. (**d**) Control sample cDNA sequencing result. (**e**) The frameshift observed in the proband’s cDNA sequencing results is caused by a 16-base deletion. In the diagram, sequences belonging to two overlapping transcripts are shown as Transcript 1 and Transcript 2. The ‘…’ symbol indicates that the sequence of bases continues in an ordered manner. The proband is indicated by a black arrow in the figure. The red arrows in the figure point to the bases with their respective base numbers labeled. CRC: colorectal cancer; FAP: familial adenomatous polyposis; LEU: leukemia; LYMPH: lymphoma; NSCLC: non-small cell lung cancer.

Proband 1 (P1) was a 48-year-old female patient diagnosed with colorectal cancer. Her family history included first-degree relatives with colorectal cancer (48 y and 55 y), colorectal and lung cancer (30 y), and familial adenomatous polyposis (40 y). Additionally, second-degree relatives had been diagnosed with leukemia (4 y), lymphoma (65 y), and colorectal cancer (54 y) Figure 1a) (Table 2). Due to her family history and diagnosis, the patient underwent genetic testing at our clinic, where the *APC* c.532-1G>A rs1554072547 (48.3%) variant was identified. Given this variant’s rarity and critical location, further investigation was warranted.

This variant was not present in the gnomAD population database [25] and was only observed once among our tests. Additionally, it was detected 3 times out of 375,000 tests (0.0008%) analyzed using the Sophia DDM software (version 5.10, Sophia Genetics, Switzerland). MutationTaster (v.2021) predicted the variant to be pathogenic, while ESEfinder 3.0 and RESCUE-ESE tools(accessed via Alamut Visual Plus Software, 2024) predicted no significant splicing alteration [16,17,26].

To evaluate the effect of this variant on mRNA splicing, we amplified the relevant gene region using RT-PCR, followed by Sanger sequencing, and compared the results to the reference sequence (Figure 1b,c). Sequencing revealed a 16-base pair deletion at the start of exon 6 (Figure 1d), corresponding to the r.532_547del mutation. This deletion is expected to cause a frameshift, resulting in a premature stop codon at p.(Phe178Ilefs*2). This premature stop codon may trigger Nonsense-Mediated Decay (NMD), potentially leading to the degradation of the transcript and a reduction in the expression of the truncated protein. Notably, this is the first time this intronic variant has been shown to cause a 16-base pair deletion, which is reported in this study. Sequencing of a control sample showed a normal result consistent with the reference sequence (Figure 1e).

The *APC* c.532-1G>A has been classified as “Pathogenic/Likely Pathogenic” in the ClinVar database [27] and is also categorized as a “Disease Causing Mutation” in the HGMD database [22]. Initially described by Wallis et al. in 1999 [28], subsequent studies have supported its pathogenicity, including potential splice errors, as evaluated using the American College of Medical Genetics Criteria (ACMG) [29]. However, no functional studies have provided direct evidence regarding this variant until now [30,31].

In conclusion, our findings demonstrate that the *APC* c.532-1G>A variant is associated with a 16-base deletion, providing novel functional evidence of its pathogenicity. This discovery contributes to a better understanding of the variants’ clinical significance and has important implications for further research and clinical practice, particularly in explaining the patients’ family history and diagnosis.

### 3.2. BLM c.4076+4T>G rs183176301

**Figure 2:**
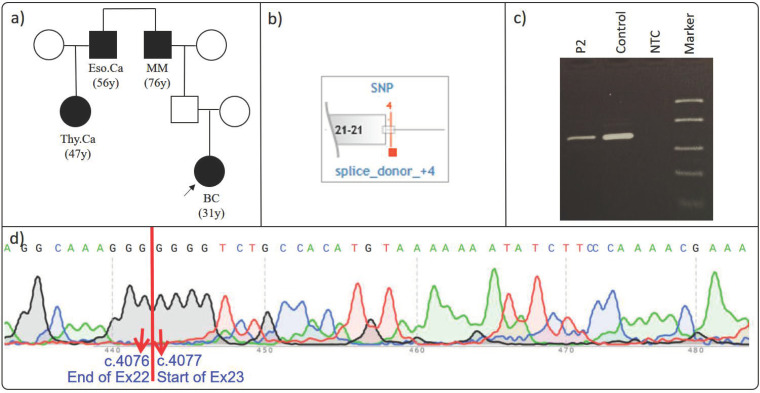
(**a**–**d**): Effect of *BLM* c.4076+4T>G on splicing. (**a**) Proband’s pedigree. (**b**) Diagram showing the variant’s proximity to the relative exon and its location. (**c**) RT-PCR agarose gel electrophoresis: the relevant region was successfully amplified on proband’s cDNA. (**d**) Proband’s Sanger sequencing result: the cDNA sequencing result of the patient’s sample was consistent with the reference gene sequence. The proband is indicated by a black arrow in the figure. The red arrows in the figure point to the bases with their respective base numbers labeled. BC: breast cancer; Eso. Ca: esophageal cancer; Thy. Ca: thyroid cancer; MM: Malign Melanoma.

Proband P2, a 31-year-old woman diagnosed with breast cancer, had a family history of skin cancer (76 y) in a second-degree paternal relative, esophageal cancer (56 y) in a third-degree paternal relative, and thyroid cancer (47 y) in a fourth-degree paternal relative (Figure 2) (Table 2). Due to her family history and early age of diagnosis, the patient underwent genetic testing, which revealed the *BLM* c.4076+4T>G (VF: 50.7%) variant. This variant, which was located near the exon 22 and potentially within the splice donor region of the *BLM* gene, attracted attention due to its rarity and its location in a region susceptible to splicing errors. It is observed in approximately 1% of the 167,000 tests analyzed by the Sophia DDM software (version 5.10, Sophia Genetics, Switzerland), and the gnomAD frequency of the variant is 0.0042 [25].

Although the HGMD database associates this variant with breast cancer as a “Disease Causing Mutation”, it is classified as benign in the ClinVar database [32]. Predictions using tools such as ESEfinder and RESCUE-ESE did not indicate any significant risk [16,17]. Given the lack of functional analysis in previous studies, we proceeded with our investigation.

To evaluate the variant’s impact on mRNA splicing, we performed RT-PCR (Figure 2), followed by Sanger sequencing of the cDNA. The sequencing results showed that the transition between exon 22 and exon 23 was normal, indicating that the identified variant did not lead to a splicing error (Figure 2). While Guacci et al. reported insufficient information for the classification of the variant [33], Tsaousis et al. classified it as a Variant of Uncertain Significance (VUS) in their study [34]. Our results for *BLM* c.4076+4T>G may help to resolve these uncertainties and suggest that this variant could be re-classified as benign.

### 3.3. BRCA1 c.4358-3A>G rs1567779966

**Figure 3:**
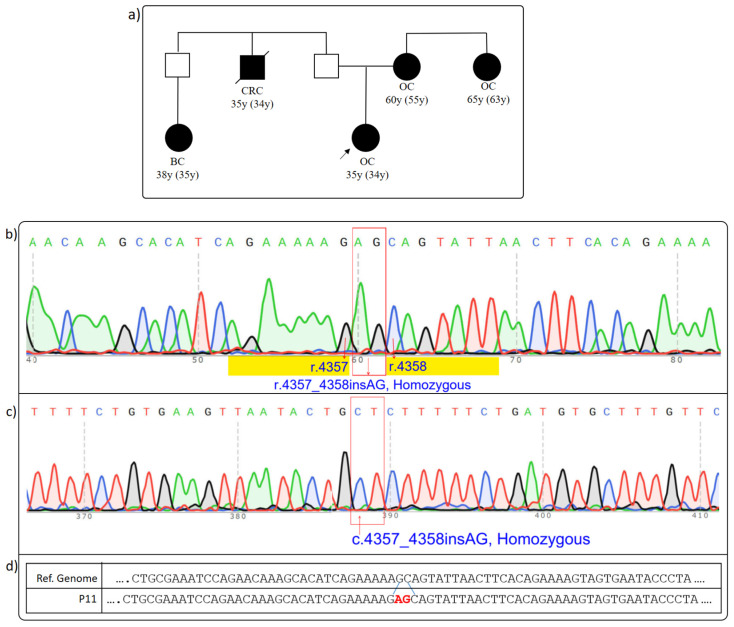
(**a**–**d**): Effect of *BRCA1* c.4358-3A>G on splicing. (**a**) Proband’s pedigree. (**b**) Proband’s Sanger sequencing result (forward sequencing): two bases are homozygously inserted compared to the reference gene sequence. (**c**) Patient’s Sanger sequencing result (reverse sequencing): the two-base insertion is observed homozygously again. (**d**) Schematic representation of the inserted bases on the reference sequence. The proband is indicated by a black arrow in the figure. The red arrows in the figure point to the bases with their respective base numbers labeled. The red rectangles indicate the inserted AG bases. BC: breast cancer; CRC: colorectal cancer; OC: ovarian cancer.

Proband 3, a 34-year-old patient diagnosed with ovarian cancer, was referred to our clinic for genetic testing due to the early age of diagnosis and significant family history. The proband’s family history included ovarian cancer (55 y) in a first-degree relative, ovarian cancer (63 y) in a second-degree maternal relative, colorectal cancer (34 y) in a second-degree paternal relative, and breast cancer (35 y) in a third-degree paternal relative (Figure 3a) (Table 2).

The *BRCA1* c.4358-3A>G variant (VF: 49.6%) was identified in the proband. This variant has not been previously reported in the gnomAD population database [25] and was observed only 10 times out of 493.000 tests analyzed by the Sophia DDM software (version 5.10, Sophia Genetics, Switzerland). Predictions from Rescue-ESE and ESE-Finder tools indicated a potential risk of splicing errors, prompting further investigation [16,17]. It is important to note that when the AG insertion was analyzed using the Alamut^TM^Visual Plus (version 1.6.1), the program assumed that the AG bases were inserted into the intron rather than the exon, which impacted the accuracy of its calculations. In reality, the AG bases are inserted into the exon, leading to a frameshift and premature stop codon formation.

RT-PCR and sequencing revealed a 2-base insertion (“AG”) between r.4357 and r.4358, corresponding to the junction between the last base of the 13th exon and the first base of the 14th exon (Figure 3b–d). This AG insertion is expected to cause a frameshift, resulting in a premature stop codon (TAG) at p.1461, beginning from the altered p.1456 amino acid position. The formation of this premature stop codon may activate Nonsense-Mediated Decay (NMD), potentially leading to the degradation of the aberrant transcript and preventing the production of the truncated protein. Although this variant has not been recorded in the ClinVAR database [23], it is listed in the HGMD database as “Disease Causing Mutation?” (Accession Number: CS199298), with references to two publications. Pirim et al. suggested that this variant may reside in a regulatory splice site and potentially affect alternative splicing, while Tsaousis et al. classified it as a Variant of Uncertain Significance (VUS) [34,35]. Additionally, the German Consortium for Hereditary Breast and Ovarian Cancer at University Hospital Cologne noted in the ClinVar Miner database that RNA analysis was warranted for this variant, classifying it as PM2 (Supporting Pathogenic). This adds further evidence in support of its pathogenicity. Our study is the first to present RNA analysis results for this variant, directly confirming its impact on the splice-site region through sequencing. Our experimental evidence suggests that this variant could be reclassified from VUS to Pathogenic, given the significant alterations it induces in mRNA structure and splicing.

In conclusion, the *BRCA1* c.4358-3A>G variant highlights the importance of integrating computational predictions with experimental data to accurately assess its pathogenicity. The identification of a frameshift mutation and its impact on protein structure underscore the clinical significance of this variant in the context of cancer predisposition.

### 3.4. BRCA2 c.7436-1G>C rs886040939

**Figure 4:**
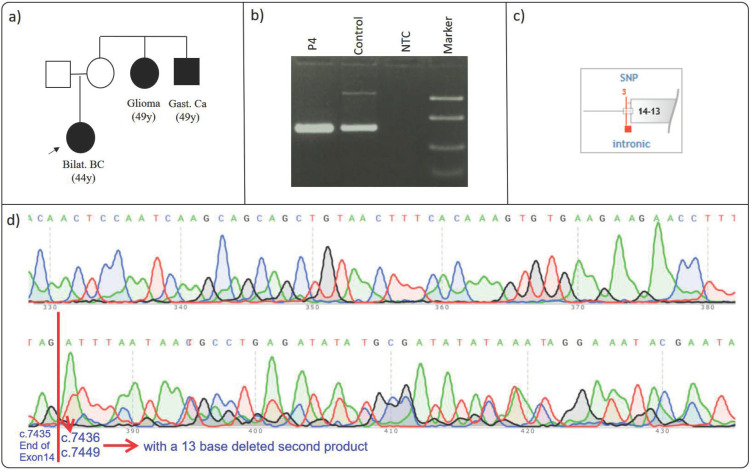

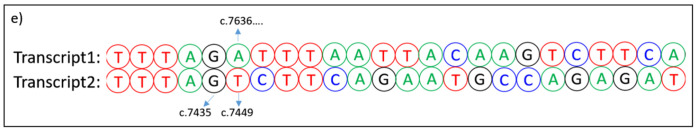
(**a**–**e**): Effect of *BRCA2* c.7436-1G>C on splicing. (**a**) Proband’s pedigree. (**b**) RT-PCR agarose gel electrophoresis: the relevant region was amplified from the proband’s cDNA. (**c**) Diagram showing the variant’s proximity to the relative exon and its location. (**d**) Proband’s Sanger sequencing result: the frameshift observed in the proband’s sequencing results is caused by a 13-bases heterozygous deletion. (**e**) In the diagram, sequences belonging to two overlapping transcripts are shown as Transcript 1 and Transcript 2. The proband is indicated by an arrow in the figure. The ‘…’ symbol indicates that the sequence of bases continues in an ordered manner. Other arrows in the figure point to the bases with their respective base numbers labeled. Bilat. BC: bilateral breast cancer; Gast. Ca: gastric cancer.

Proband P4 was diagnosed with early-onset bilateral breast cancer (44 y) and had a family history of glioma (49 y) and gastric cancer (49 y) in maternal second-degree relatives (Figure 4a) (Table 2). Genetic testing revealed an intronic missense alteration, c.7436-1G>C (VF:49.3%), in the *BRCA2* gene of the proband. To evaluate its clinical significance of the c.7436-1G>C variant, we conducted a comprehensive examination. Sequencing results revealed a 13-base pair deletion at the start of *BRCA2* exon 15 (Figure 4b,c), leading to a frameshift that transformed the amino acid sequence at position p.(Asp2478Val) and introduced a premature stop codon.

This variant, notable for its rarity and critical location, has been classified as “Pathogenic” in both the ClinVAR and HGMD databases [22,36]. However, no prior studies have provided functional analysis to confirm its pathogenicity. Pirim et al. suggested that the variant may be pathogenic due to its position within the splice region [35].

Our results showed a heterozygous pattern, with two forms of exon 15 observed: the normal exon 15 and the altered form with the first 13 bases deleted. This deletion caused a frameshift, resulting in the substitution of Asparagine at position 2478 with Valine, and subsequently leading to the formation of a premature stop codon (TGA) at amino acid 2518. Consequently, the presence of the c.7436-1G>C variant is predicted to result in the production of a truncated *BRCA2* protein, further supporting its pathogenic classification.

### 3.5. MSH3 c.1897-1G>A rs1744149615

**Figure 5:**
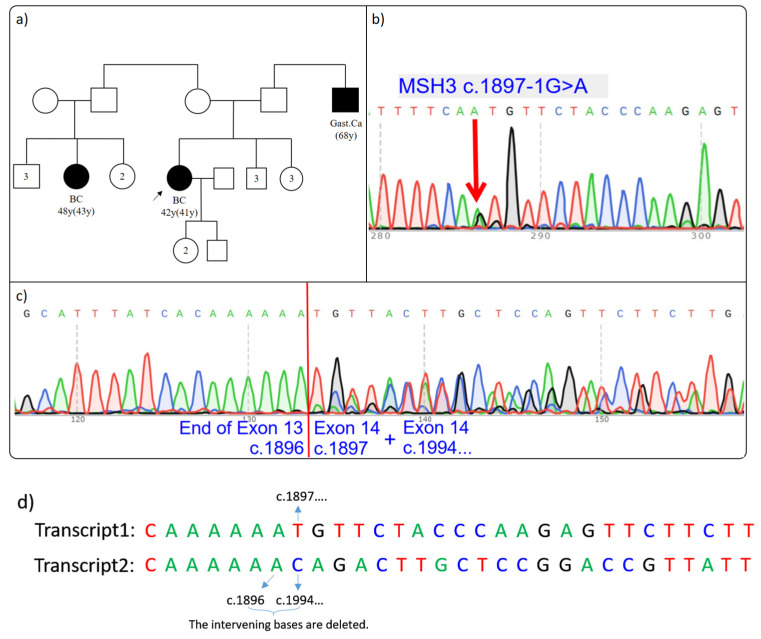
(**a**–**d**): Effect of MSH3 c.1897-1G>A on splicing. (**a**) Proband’s pedigree. (**b**) The presence of the variant on proband’s genomic DNA: Sanger sequencing results for genomic DNA are consistent with the NGS results. (**c**) Patient’s RT-PCR Sanger sequencing result: partially heterozygous deletion of exon 14. (**d**) In the diagram, sequences belonging to two overlapping transcripts are shown as Transcript 1 and Transcript 2. The proband is indicated by an arrow in the figure. BC: breast cancer; Gast. Ca: gastric cancer.

Proband P5, a 42-year-old female diagnosed with breast cancer, had a family history that included gastric cancer (68 y) in a second-degree paternal relative and breast cancer 48 y (43 y) in a third-degree maternal relative (Figure 5a) (Table 2). Due to her early age diagnosis, the patient underwent genetic testing at our clinic, which revealed the *MSH3* c.1897-1G>A rs1744149615 variant (VF: 48.0%). This variant was not present in the gnomAD population database [25] and was observed only once among our tests. Additionally, it was detected in only 0.008% of tests analyzed by the Sophia DDM software (version 5.10, Sophia Genetics, Switzerland). MutationTaster analysis suggested potential pathogenicity for this variant [26].

To investigate the impact of this variant on mRNA splicing, sequencing was performed on both the patient’s DNA and cDNA (Figure 5b). Results revealed a 97-base pair deletion between r.1897 and r.1993, along with a 1-base insertion “A” at the start of exon 14 (Figure 5c). The c.1897-1G>A variant disrupts the 3′ splice acceptor AG pattern, impacting splicing at this canonical site. This inserted “A” base likely represents the first base of the intron, introducing a new splice site that leads to the removal of nearly half of exon 14 from the *MSH3* gene. However, the 1-base insertion after the deletion compensates for the missing base of the 33rd amino acid, maintaining the reading frame. Consequently, we anticipate the formation of the p.(Cys633_Leu645delinsIle) transcript, as the 97-base pair deletion and A insertion result in an in-frame alteration that avoids the introduction of a premature stop codon.

In the ClinVAR database, the variant is labeled as “Likely Pathogenic” based on in silico predictions despite the absence of functional evidence [37]. No relevant data were found in the HGMD database [22]. Furthermore, the ClinVar Miner database includes a note from other researchers suggesting that this variant is likely pathogenic due to canonical splice-site disruption. Our results align with this assessment and provide the first RNA-based evidence of the variant’s effect on splicing. Although the rarity of this variant in patients suggests a potential loss of protein function due to the deleted amino acids, further research is required to fully understand its impact. Our study is the first to demonstrate the functional consequences of this variant.

### 3.6. RB1 c.2489+2T>C rs1555294636

**Figure 6:**
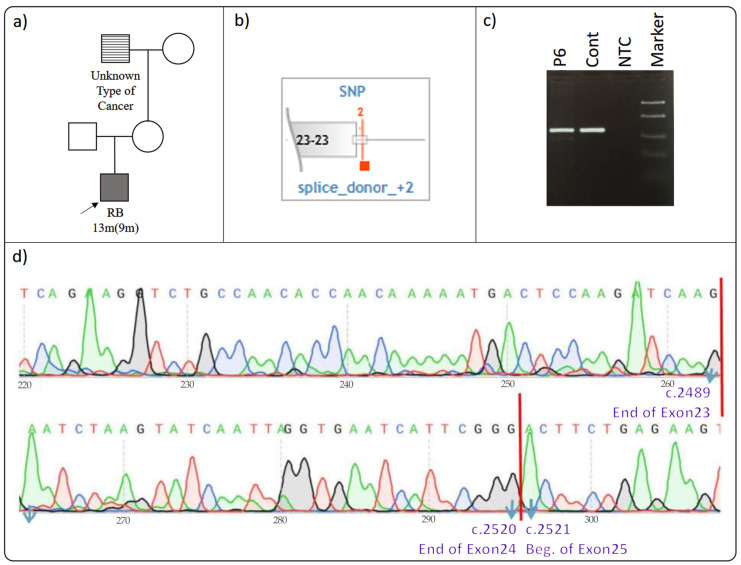
(**a**–**d**): Effect of *RB1* c.2489+2T>C on splicing. (**a**) Proband’s pedigree. (**b**) Diagram showing the variant’s proximity to the relative exon and its location. (**c**) RT-PCR agarose gel electrophoresis. (**d**) Patient’s Sanger sequencing result: the cDNA sequencing result of the proband’s sample was consistent with the reference gene sequence. The proband is indicated by an arrow in the figure. The other arrows in the figure point to the bases with their respective base numbers labeled. RB: retinoblastoma.

Proband P6 was diagnosed with retinoblastoma 13 months (9 months) and had a second-degree maternal relative with an unspecified cancer diagnosis (Figure 6a) (Table 2). The patient was referred to our cancer genetics clinic for *RB1* gene analysis due to retinoblastoma diagnosis. Comprehensive gene screening using NGS revealed the presence of the rare *RB1* c.2489+2T>C variant (VF:48.7%), which had never been observed in the general population. This variant was identified for the first time among 158,000 tests analyzed in the Sophia DDM software (version 5.10, Sophia Genetics, Switzerland). In silico tools predicted that the variant, located at the splice donor site, could result in a splicing error (Figure 6b) [16,17].

To assess the impact of this variant on mRNA, sequencing was performed following RT-PCR. Electrophoresis of the amplified products on an agarose gel revealed bands of identical size in both patient and control samples, suggesting no size differences (Figure 6c). Subsequent sequencing confirmed the normal arrangement of the 23rd, 24th, and 25th exons, with no splicing errors induced by the c.2489+2T>C variant (Figure 6d).

The *RB1* c.2489+2T>C variant, also referred to as *RB1* IVS23+2T>C, is not listed in the HGMD database [22], but it is classified as “Pathogenic” in the ClinVAR database, supported by seven publications as evidence [38]. However, five of these studies referenced IVS23+1 instead of IVS23+2, likely due to the proximity of the c.2489+2T>C variant to c.2489+1, leading researchers to treat them as the same variant. Only one publication provided insights into splicing mechanisms [39], while another applied ACMG criteria to assess its clinical significance [40]. The primary variant discussed in these publications is c.2489+1, but the variant investigated in the study is c.2489+2 [31,32,33,34,35]. Current evidence does not strongly support the idea that this variant causes a splicing error as previously suggested.

Given the collective evidence, we question the pathogenic classification of the c.2489+2T>C variant in the ClinVar database [38]. Our findings indicate that it does not exhibit pathogenic effects in patient samples, raising doubts about its classification. Further research and functional studies are needed to clarify its exact role in *RB1* gene splicing and its relationship with the observed clinical phenotype.

### 3.7. RB1 c.1050-2 A>G rs?

**Figure 7:**
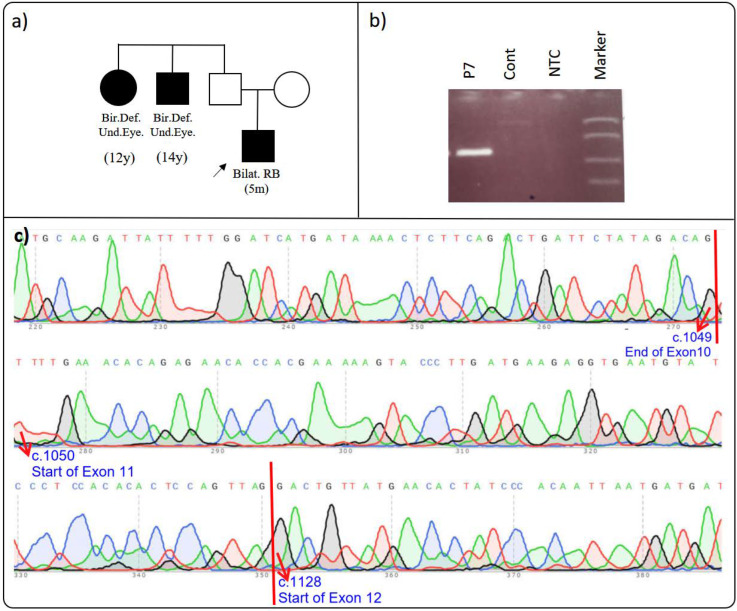
(**a**–**c**): Effect of *RB1* c.1050-2A>G on splicing. (**a**) Proband’s pedigree. (**b**) RT-PCR agarose gel electrophoresis: the relevant region was successfully amplified from the proband’s cDNA. (**c**) Proband’s Sanger sequencing result: the cDNA sequencing result of the patient’s sample was consistent with the reference gene sequence. The proband is indicated by an arrow in the figure. The red arrows in the figure point to the bases with their respective base numbers labeled. Bir. Def. Und. Eye: birth defect, undeveloped eyeballs; Bilat. RB: bilateral retinoblastoma.

Proband P7 was diagnosed with bilateral retinoblastoma at five months. Upon reviewing the family history, it was noted that two second-degree relatives, currently aged 12 and 14, were born with undeveloped eyeballs, possibly indicating microphthalmia (Figure 7a) (Table 2). Genetic testing revealed the *RB1* c.1050-2A>G variant (VF: 48.9%) in the proband. This variant, located at the beginning of exon 11 and potentially within the splice acceptor region, is rare and was found only once among 158,000 tests (0.00063%) analyzed by the Sophia DDM software (version 5.10, Sophia Genetics, Switzerland). Several computational tools, including MutationTaster, SpliceSiteFinder, MaxEntScan, NNSPLICE, GeneSplicer, and Human Splicing Finder, predict it to be “Deleterious” and likely to cause splicing errors [12,13,15,26,41]. The Sophia DDM software also classified it as pathogenic, and it has been cited in the LOVD and HGMD databases [22,24]. However, it is important to note that a publication by Shimizu et al. [42] mentioned only the c.1050-2A>G variant without explicitly stating its effect on the amino acid sequence. Additionally, Li et al. reported the c.1050-2A>T variant in a patient with retinoblastoma in 2022, suggesting that this variant may lead to a change in the splice-site region [43]. These findings underscore the complexity of variant interpretation and the limitations of relying solely on computational tools, emphasizing the need for experimental validation.

To assess the variant’s effect on transcript formation, we amplified and sequenced the relevant gene region (Figure 7b). Surprisingly, we found that the variant did not cause splicing errors, with correct splicing of exons 9-10-11-12 in the patient’s RNA (Figure 7c). This result suggests the need for a re-evaluation of the variant’s pathogenicity.

In conclusion, the *RB1* c.1050-2A>G variant underscores the importance of combining computational predictions with experimental data for the comprehensive evaluation of its potential pathogenicity. This approach is critical for accurate variant interpretation and clinical decision-making in genetic disorders.

## 4. Conclusions

In conclusion, this study highlights the pivotal role of RNA splicing in inherited cancer predisposition. We have demonstrated the potential pathogenicity of certain variants by examining specific variants within cancer-related genes and their effects on RNA splicing patterns in cancer patients. Notably, the identification of four pathogenic variants, *APC* c.532-1G>A rs1554072547, *BRCA1* c.4358-3A>G rs1567779966, *BRCA2* c.7436-1G>C rs81002830, and *MSH3* c.1897-1G>A rs1744149615, underscores the critical importance of understanding the consequences of splice-site variants through functional studies. Additionally, we observed that some variants, such as *BLM* c.4076+4T>G rs183176301, *RB1* c.2489+2T>C rs1555294636, and *RB1* c.1050-2A>G rs?, despite being predicted as pathogenic based on location, did not exhibit significant effects.

These findings emphasize the need for further investigation into the mechanisms and functional implications of mRNA splicing, particularly in the context of genetic disorders and cancer predisposition. Understanding splice-site variants in hereditary cancer is crucial for accurate diagnosis and holds potential implications for developing personalized therapeutic strategies. Given the increasing interest in RNA-based therapeutic approaches, modified snRNA and antisense oligonucleotide therapies are emerging as promising strategies to address splicing-related defects. These RNA-based therapies could potentially correct splicing errors, reduce the effects of pathogenic splicing alterations, and provide personalized intervention options for individuals with hereditary cancer predisposition. Moreover, this study contributes to the clinical management of patients by helping to identify individuals who may benefit from enhanced surveillance or tailored therapeutic interventions based on their genetic risk. Confirming pathogenic splicing variants can provide more accurate genetic counseling for families, allowing for better-informed decisions about preventive measures or treatment options.

This research may facilitate a more precise understanding of splice-site variants, guiding the development of patient-specific management plans and improving outcomes for those with inherited cancer risks. Continued research in this domain is crucial for identifying potential therapeutic targets related to cancer-related aberrant splicing events. This study contributes to a deeper understanding of the impact of splice-site variants on inherited cancer susceptibility, underscoring the importance of ongoing research into therapeutic strategies that stem from a more nuanced understanding of splicing mechanisms in cancer.

## Figures and Tables

**Table 1:** Primer sequences used for RT-PCR and Sanger sequencing in the study.

Proband	Reference	Gene and Variant	rs number	F/R?	Exon	Primer Location	Primer Sequence
P1	NM_000038.4	*APC* c.532-1G>C	rs1554072547	F Primer	Exon 4	c.281_c.300	5′-GTTCTTATGGAAGCCGGGAA-3′
				R Primer	Exon 8	c.805_c.824	5′-CCAGAAGTTGCCATGTTGAT-3′
P2	NM_000057	*BLM* c.4076+4T>G	rs183176301	F Primer	Exon19	c.3620_3639	5′-AAGTGTCTCAGAGGGAAGAGA-3′
				R Primer	Exon 22	c.4192_c.4220	5′-GCTTCGGTGGAGCCATAAT-3′
P3	NM_007294.3	*BRCA1* c.4358-3A>G	rs1567779966	F Primer	Exon 13	c.4271_4294	5′-AGCCTTCTAACAGCTACCCTTCCA-3′
				R Primer	Exon 16	c.4742_c.4761	5′-TGACTCTGGGGCTCTGTCTT-3′
P4	NM_000059.3	*BRCA2* c.7436-1G>C	rs81002830	F Primer	Exon 14	c.7021_c.7041	5′-CGTCAAGAGATACAGAATCC-3′
				R Primer	Exon 16	c.7620_c.7640	5′-CAGCTGTTTATGAGAACACG-3′
P5	NM_002439.4	*MSH3* c.1897-1G>A	rs1744149615	F Primer	Exon 12	c.1740_c.1760	5′-GACCCAGCCACTCCTTAAAT-3′
				R Primer	Exon 15	c.2164_c.2186	5′-TGCATTCGGATCTCGTCAATAA-3′
P6	NM_000321.2	*RB1* c.2489+2T>C	rs1555294636	F Primer	Exon 21	c.2190_c.2211	5′-TCTTCCTCATGCTGTTCAGGAG-3′
				R Primer	Exon 25	c.2639_c.2660	5′-CCATCTGCTTCATCTGATCCTT-3′
P7	NM_000321.2	*RB1* c.1050-2A>G	?	F Primer	Exon5–6	c.529_c.548	5′-ATCGAACTGCTGGGTGTGT-3′
				R Primer	Exon13	c.1329_c.1348	5′-CTCCAAGTTTGTATCGCTGT-3′

The table lists the forward and reverse primer sequences synthesized for the amplification of specific regions in each gene, used in both RT-PCR and Sanger sequencing analyses.

**Table 2:** Patient characteristics, gene variants, and family cancer history.

Patient	Diagnosis	Age at Diagnosis	Sex	Variant	VF%	1. Degree Cancer History	2. Degree Cancer History	3. Degree Cancer History	4. Degree Cancer History	Smoking	Alcohol History
P1	Colorectal Cancer	48 y	Female	*APC* c.532-1G>A rs1554072547	48.3%	CRC (55 y), CRC+NSCLC (30 y), FAP (40 y)	Leukemia (4 y), Lymphoma (65 y), CRC (54 y)	-	-	No	No
P2	Breast Cancer	31 y	Female	*BLM* c.4076+4T>G rs183176301	50.7%	-	Malign Melanoma (76 y)	Esophageal cancer (56 y)	Thyroid cancer (47 y)	Yes	No
P3	Ovarian Cancer	34 y	Female	*BRCA1* c.4358-3A>G rs1567779966	49.6%	Ovarian Cancer (55 y)	Ovarian cancer (63 y), Colorectal cancer (34 y)	Breast cancer (35 y)	-	No	No
P4	Bilateral Breast Cancer	44 y	Female	*BRCA2* c.7436-1G>C rs886040939	49.3%	-	Glioma (49 y), Gastric Cancer (49 y)	-	-	No	No
P5	Gastric Cancer	42 y	Female	*MSH3* c.1897-1G>A rs1744149615	48.0%	-	Gastric cancer (68 y)	Breast cancer 48 y (43 y)	-	No	No
P6	Retinoblastoma	9 m	Male	*RB1* c.2489+2T>C rs1555294636	48.7%	-	Unspecified cancer diagnosis at one relative	-	-	No/NA	No/NA
P7	Retinoblastoma	5 m	Male	*RB1* c.1050-2A>G rs?	48.9%	-	Two children born with underdeveloped eyeballs	-	-	No/NA	No/NA

Summary of patient characteristics, including diagnosis, age at diagnosis, sex, specific gene variants, variant frequencies (VF%), and family cancer history across first- to fourth-degree relatives. Smoking and alcohol history is also provided for each patient.

## Data Availability

The original contributions presented in this study are included in the article/Supplementary Material, further inquiries can be directed to the corresponding author/s.

## Supplementary Materials

The following supporting information can be downloaded at: https://www.mdpi.com/article/10.3390/cimb46110790/s1. Table S1: Bioinformatic and In Silico Analysis Information.
